# Prevalence of human papillomavirus in sinonasal squamous cell carcinoma with and without association of inverted papilloma in Eastern China

**DOI:** 10.1186/s13027-020-00298-4

**Published:** 2020-05-29

**Authors:** Chunyan Hu, Huatao Quan, Li Yan, Ji Sun, Lin Lan, Shengzi Wang

**Affiliations:** 1 Department of Pathology, Eye and ENT Hospital, Fudan University, Shanghai, China; 2 Department of Radiation Oncology, Eye and ENT Hospital, Fudan University, Fenyang Road 83, Xuhui District, Shanghai, China

**Keywords:** Sinonasal carcinoma, Squamous cell carcinoma, HPV, Inverted papilloma

## Abstract

**Background:**

Information on HPV-associated sinonasal squamous cell carcinoma (SNSCC) is very limited in China. The aim of this study was to determine the prevalence of HPV in a large cohort of SNSCC patients in China.

**Methods:**

Clinical records and formalin-fixed and paraffin-embedded tumor specimens from 30 SNSCC patients with associated inverted papilloma (IP-SNSCC) and 84 de novo SNSCC (DN-SNSCC) patients were retrieved between 2010 and 2017. HPV status was determined for each specimen using a combination of p16 immunohistochemistry and GP5+/6+ PCR.

**Results:**

Immunohistochemistry for p16 was positive in two IP-SNSCC patients (2/30, 6.7%) and in 16 DN-SNSCC patients (16/84, 19.0%). HPV DNA was detected in six IP-SNSCC patients (6/30, 20%) and in three DN-SNSCC patients (3/84, 3.8%). Expression of p16 was not correlated with the presence of HPV DNA (*p* = 0.150). Among 18 p16-positive SNSCC patients, only three were HPV DNA-positive. Furthermore, only three of nine HPV DNA-positive tumors exhibited high p16 expression. In IP-SNSCC patients, only one of six HPV DNA-positive tumors exhibited high p16 expression. In DN-SNSCC patients, two of three HPV DNA-positive tumors exhibited high p16 expression. The positive rates for both HPV DNA and p16 in IP-SNSCC patients and DN-SNSCC patients were 3.3 and 2.4%, respectively.

**Conclusions:**

Immunostaining for p16 is not a reliable surrogate marker of HPV status in SNSCC. The presence of HPV is rarely detected in DN-SNSCC patients in Eastern China. IP-SNSCC patients frequently lack of p16 overexpression despite the presence of high-risk HPV DNA.

## Background

Infection with human papillomavirus (HPV), particularly high-risk types of HPV, has been acknowledged as an etiological factor in a subset of head and neck cancers [1]. Head and neck squamous cell carcinomas (SCCs) caused by HPV arise predominantly in the oropharynx [2]. HPV-positive oropharyngeal SCC has unique clinicopathological features and an improved prognosis when compared to HPV-negative oropharyngeal SCC [3]. However, the clinical significance of HPV infection in non-oropharyngeal head and neck SCCs remains uncertain [4].

The presence of HPV has also been reported in SCCs of the sinonasal tract, but HPV detection rates in sinonasal SCC (SNSCC) patients are widely disparate and range from 0 to 100% [5]. There are various reasons for these inconsistent detection rates. First, the prevalence of HPV varies with ethnicity and geographic origin [3, 6]. Second, most previous studies used dissimilar HPV detection methods and small sample sizes [5]. Lastly, many studies have failed to distinguish between de novo sinonasal SCC (DN-SNSCC) and SNSCC arising from inverted papillomas (IP-SNSCC).

Inverted papilloma (IP) is the most common benign tumor of the sinonasal tract, with a 5–13% incidence malignant transformation [7]. The most common associated malignancy is SCC [8]. Although the development of IP is not fully understood, some studies have suggested HPV acts as a potential etiological agent in the progression of IP to SCC [9, 10].

To date, the clinical significance of HPV status in SNSCC is not well established and information on HPV-associated SNSCC in China is very limited. Here, we analyzed the presence of HPV infection in DN-SNSCC and IP-SNSCC patients in a large Eastern China population.

## Materials and methods

### Patients

Formalin-fixed and paraffin-embedded (FFPE) tissues were retrospectively collected from SNSCC patients who underwent surgical treatment at the Eye and ENT Hospital of Fudan University between 2010 and 2017. Hematoxylin and eosin-stained sections were reviewed and histological diagnosis was confirmed by an experienced head and neck pathologist. FFPE tissues without tumor cells were excluded. In this study, specimens from 30 IP-SNSCC patients and 84 DN-SNSCC patients were obtained for HPV detection. Clinical information was extracted from medical records. Tumor node metastasis (TNM) stage was assessed according to the 7th edition of the American Joint Committee on Cancer staging system.

### Ethics declarations

The study was approved by the Institutional Review Committee of the Eye and ENT Hospital of Fudan University. Informed consent was obtained from all patients. All procedures were conducted in accordance with the Declaration of Helsinki.

### p16 immunohistochemistry

Immunohistochemical analysis for p16 was performed on 4-μm thick tissue sections cut from the FFPE tissue. A monoclonal antibody to p16 (ab108349, Abcam, UK; dilution 1:100) was used with a BenchMark Autostainer (Ventana Medical Systems, Tucson, USA) following the manufacturer’s recommendations. Expression of p16 was considered positive if strong and diffuse nuclear and cytoplasmic staining was detected in ≥70% of the tumor [11] Two head and neck pathologists reviewed p16 expression and were blinded to the clinical and follow-up data. Discrepancies that occurred between observers were resolved by consensus review.

### Extraction of DNA

FFPE tissues were cut into five 10-μm thick sections on glass slides, and DNA was extracted using a commercial kit (TIANamp FFPE DNA Kit, China) according to the manufacturer’s instructions. The quantity and quality of extracted DNA was determined via NanoDrop ND-1000 spectrophotometry (Thermo Fisher Scientific Inc., Waltham, MA, USA). Isolated DNA was stored at − 20 °C until further use.

### HPV DNA detection and genotyping

HPV DNA detection was carried out utilizing general primers GP5+/6+. Validation of the DNA quality and efficacy of the PCR was assessed using the housekeeping gene β-globin. DNA from HPV-positive uterine cervix tissue was used as a positive control and reactions without a template were run in parallel as controls. The amplified products were subjected to electrophoresis in 2% agarose gels and observed by nucleic acid dye. Tissues positive for HPV DNA were then further analyzed using a commercial HPV genotyping kit (Chaozhou Hybribio Limited Corporation) [12]. This kit can identify 23 HPV genotypes, including 17 high-risk types (HPV 16, 18, 31, 33, 35, 39, 45, 51, 52, 56, 58, 59, 66, 68, 53, 73, 82) and six low-risk types (HPV 6, 11, 42, 43, 44, 81).

### Statistical analysis

All statistical analyses in this study were performed using SPSS 22.0 software (IBM Corp, Armonk, NY). The chi-squared test or Fisher’s exact test were used to analyze categorical variables. The student’s t-test or Mann-Whitney U tests were used to analyze continuous variables according to application conditions. All *p*-values were two-sided, and p-values < 0.05 were considered statistically significant.

## Results

### Patient characteristics

In our study, 94.7% (108/114) of patients were from Eastern China and were mainly distributed in Shanghai, Zhejiang province, Jiangxi province, Jiangsu province, and Anhui province. The remaining patients were from other regions of China. The clinicopathologic data of patients are listed in Table 1. The median patient age for the IP-SNSCC and DN-SNSCC groups was 58 and 59 years, respectively. Both the IP-SNSCC and DN-SNSCC groups presented with male predilection (73.3 and 73.8%, respectively). A smoking history was reported in 33.3% of IP-SNSCC patients and 39.3% of DN-SNSCC patients. Most of the patients in our study were in the T3-T4 stage. No patient had distant metastases at the time of diagnosis. There were no differences in age, sex, smoking history, tumor primary site, or disease stage between IP-SNSCC and DN-SNSCC patients (Table 1).

**Table 1:** Clinical characteristics of patients with IP-SNSCC and DN-SNSCC

Characteristics	IP-SNSCC (***n*** = 30)	DN-SNSCC (***n*** = 84)	P
Age, in years [Median (range)]	58 (45–82)	59 (24–84)	0.654
Sex, no. (%)			
Male	22 (73.3)	62 (73.8)	0.959
Female	8 (26.7)	22 (26.2)	
Smoking history, no. (%)			
Yes	10 (33.3)	33 (39.3)	0.564
No	20 (66.7)	51 (60.7)	
Primary site, no. (%)			
Nasal cavity	21(70.0)	57 (67.9)	0.828
Paranasal sinuses	9 (30.0)	27 (32.1)	
T stage, no. (%)			
T1+ T2	2 (6.7)	9 (10.7)	0.725
T3+ T4	28 (93.3)	75 (89.3)	
N stage, no. (%)			
N0	27 (90.0)	72 (85.7)	0.759
N+	3 (10.0)	12 (14.3)	

*DN-SNSCC* de-novo sinonasal squamous cell carcinoma, *IP-SNSCC* inverted papilloma derived sinonasal squamous cell carcinoma

### Prevalence of HPV in SNSCC

**Fig. 1:**
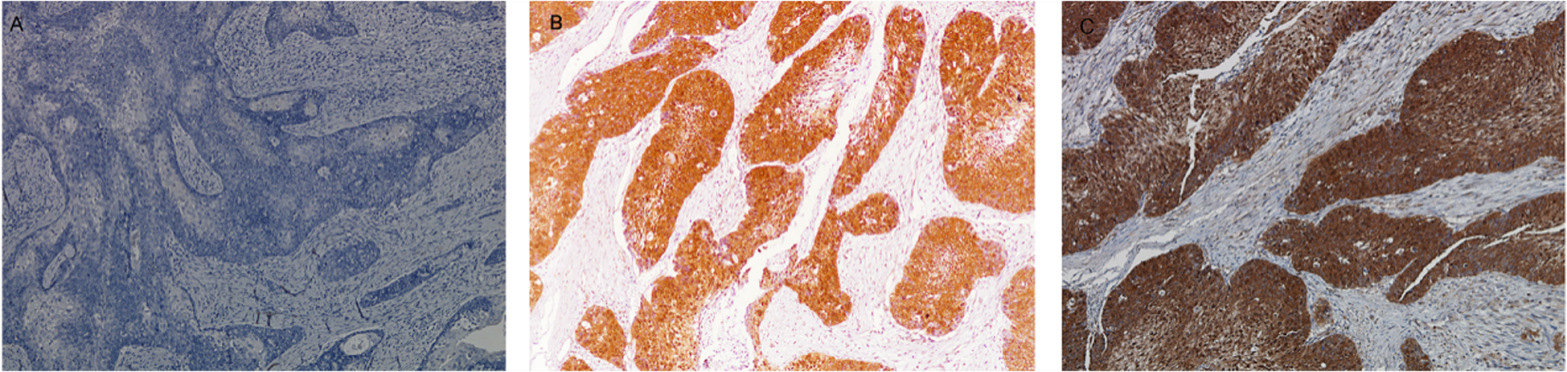
The representative immunohistochemical staining of p16 (100×). **a** p16 negative; **b** p16 positive in IP-SNSCC; **c** p16 positive in DN-SNSCC. DN-SNSCC: de-novo sinonasal squamous cell carcinoma; IP-SNSCC: inverted papilloma derived sinonasal squamous cell carcinoma

Immunohistochemistry analysis showed p16-positive tissues in two IP-SNSCC patients (2/30, 6.7%) and 16 DN-SNSCC patients (16/84, 19.0%). Representative immunohistochemical staining of p16 is shown in Fig. 1. Among 18 p16-positive SNSCC patients, HPV was detected in only three cases. HPV DNA was detected in six patients with IP-SNSCC (6/30, 20%) and in three patients with DN-SNSCC (3/84, 3.8%). Thus, the prevalence of HPV DNA in IP-SNSCC patients was significantly higher than that of DN-SNSCC patients (*p* = 0.014). In IP-SNSCC patients, only one of six HPV-positive tumors exhibited high p16 expression. The positive rates of  both HPV DNA and p16 in IP-SNSCC patients and DN-SNSCC patients were 3.3 and 2.4%, respectively. The statistical results are listed in Table 2.

**Table 2:** Prevalence of Human Papillomavirus in IP-SNSCC and DN-SNSCC

Test		IP-SNSCC (*n* = 30)	DN-SNSCC (*n* = 84)	p
p16	(+)	2(6.7%)	16(19.0%)	0.110
	(−)	28(93.3%)	68(81.0%)	
HPV DNA PCR	(+)	6(20.0%)	3(3.6%)	0.014^a^
	(−)	24(80.0%)	81(96.4%)	
p16 combine PCR	(+)	1(3.3%)	2(2.4%)	0.780
	(−)	29(96.7%)	82(97.6%)	

^a^:*p* < 0.05 *DN-SNSCC* de-novo sinonasal squamous cell carcinoma, *IP-SNSCC* inverted papilloma derived sinonasal squamous cell carcinoma

### HPV genotyping

The genotyping result of HPV-positive patients is shown in Table 3. Of the nine HPV DNA positive patients, eight were identified to have high-risk HPV-16. Only one patient had mixed infection of HPV-11 and HPV-81.

**Table 3:** clinical characteristics and genotyping of HPV DNA positive patients

Patient number	Age	Sex	Smoking	Tumor etiology	p16	HPV Type
1	57	Male	Yes	IP-SNSCC	(+)	16
2	58	Male	Yes	IP-SNSCC	(−)	16
3	49	Male	Yes	IP-SNSCC	(−)	16
4	61	Female	No	IP-SNSCC	(−)	16
5	58	Male	No	IP-SNSCC	(−)	16
6	78	Male	Yes	IP-SNSCC	(−)	16
7	55	Male	Yes	DN-SNSCC	(+)	16
8	29	Female	No	DN-SNSCC	(+)	16
9	51	Male	No	DN-SNSCC	(−)	11,81

*DN-SNSCC* de-novo sinonasal squamous cell carcinoma, *IP-SNSCC* inverted papilloma derived sinonasal squamous cell carcinoma

## Discussion

The ‘gold standard’ for transcriptionally active HPV detection in cancer patients is the analysis of HPV E6/E7 mRNA in a tumor specimen, but this becomes technically challenging when using low-quality mRNA from archival FFPE samples [13]. For FFPE specimens, commonly used HPV detection methods include HPV DNA PCR, DNA in situ hybridization, and p16 immunohistochemistry. Transcriptionally active HPV infection induces the expression of the oncogenes E6 and E7 [14]. The HPV E7 oncogene product leads to overexpression of the cyclin-dependent kinase inhibitor p16, and therefore p16 overexpression is often used as a marker of HPV infection [14]. However, p16 immunohistochemistry may lack of specificity since it does not directly detect HPV. PCR-based assays are a highly sensitive approach to detect HPV DNA, but cannot distinguish an infection that is involved in carcinogenesis from the incidental presence of virus [13]. Instead, a combination of p16 immunohistochemistry and GP5+/6+ PCR can be used to determine the HPV status with high specificity and sensitivity [15].

To the best of our knowledge, the present study is the largest study completed in China to detect the rate of HPV infection in SNSCC. The prevalence of HPV infection in SNSCC patients that was identified in our study is significantly lower than previous studies in the literature. Previous studies using strict testing methods and large samples found overall HPV infection rates of 20 to 26% in SNSCC patients [16, 17]. A retrospective study of an American population using the National Cancer Database found that the overall HPV infection rate was 31.7% in SNSCC patients [18]. These disparities are mainly due to variations in HPV prevalence across geographic origins and ethnicities. The incidence of HPV infection in head and neck cancer is high in North America and Western Europe but low in Africa and Asia [6]. Furthermore, there is a higher prevalence of HPV infection in the Caucasian race [3]. HPV prevalence in the Chinese population is lower than that of Western countries, which may be related to economic development and lifestyle [1].

HPV types are classified as high-risk or low-risk with respect to their tendency to cause malignant neoplasms. The most common high-risk type is HPV-16, which accounts for more than 80% of HPV-positive head and neck tumors [19]. Similar to previous studies, eight of nine HPV DNA-positive SNSCC patients in our study were identified to have HPV-16. The only patient identified to have low-risk HPV types (HPV-11 and HPV-81) was p16-negative. This suggests that this patient may not have an HPV-related carcinoma, since HPV DNA can also be detected in normal sinonasal mucosa. Previous studies have found that HPV-related oropharyngeal SCC patients are less likely have a history of smoking rates and are younger in age [20]. In our cohort, the limited number of HPV-positive SNSCC patients made it difficult to find a relationship between HPV status and age or smoking history.

As p16 immunohistochemistry is cost-effective and practical, it has been widely used to define HPV status. However, the association between HPV infection and p16 overexpression seems to be site-specific [21]. Although p16 is a well-established surrogate marker for HPV status in oropharyngeal SCC, the correlation between p16 and HPV is less pronounced in other tumor locations [22]. Recent studies have suggested that p16 does not have equal specificity in head and neck regions outside the oropharynx [23, 24]. A study based on reverse phase protein array data and RNA-seq found frequent overexpression of p16 protein in HPV-negative non-oropharyngeal head and neck SCC [25]. Moreover, Belobrov et al. found that p16 is not a reliable surrogate marker for HPV infection in oral SCC [24]. In our study, among the 18 p16-positive SNSCC patients, HPV DNA was identified in only three cases (3/18, 16.7%). In a recent study, Sahnana et al. found 12 SNSCC patients to be p16-positive, but only four of those 12 were HPV-positive [26]. Bishop et al. identified 26 of 127 (20.5%) HPV-negative sinonasal carcinomas that were p16-positive [16]. The high frequency of p16 overexpression in HPV-negative SNSCC demonstrates that p16 is not an accurate surrogate marker for HPV infection. The overexpression of p16 in SNSCC may be induced by non-viral mechanisms, such as genetic alteration or functional mutation [27]. Some studies have found that overexpression of p16 can be an independent favorable prognostic indicator for head and neck cancer, regardless of HPV status or tumor site [28, 29]. However, poorer survival of p16+/HPV- oropharyngeal SCC patients has also been reported [30]. In our study, we did not find a survival advantage for p16-positive SNSCC (dates are not shown in result), although the low number of p16-positive patients in our cohort may have reduced the statistical power.

HPV has been implicated in the pathogenesis of IP and high-risk HPV types appear to increase the risk of developing SCC [9]. However, very recent studies have shown that HPV is most likely not an etiological driver of IP development or progression to SCC [31, 32]. In our study, the prevalence of HPV DNA was significantly higher in IP-SNSCC patients than in DN-SNSCC patients (20% vs 3.8%, *p* = 0.014). However, only one of six HPV-positive IP-SNSCC patients exhibited high p16 expression. Lack of p16 overexpression, despite the presence of high-risk HPV DNA, indicates that viral DNA may be a bystander rather than a driver in IP-SNSCC progression. In agreement with our study, no transcriptionally active HPV has been previously reported in patients with IP-SNSCC [33]. We speculate that HPV may not play a vital role in the malignant transformation of IP.

The improved prognosis for HPV-positive oropharyngeal tumors has been well established. However, there is not sufficient evidence to demonstrate better survival rates with HPV-positive SNSCC. In our study, we were not able to analyze the association between disease prognosis and HPV status due to a limited number of HPV-positive patients. Although some previous studies have reported a better outcome for HPV-related SNSCC, others did not find a difference in survival [16, 17, 34, 35]. These investigations were all single-center retrospective studies with small sample sizes. Moreover, these studies often combined data for DN-SNSCC and IP-SNSCC patients in their findings. Some reports have described improved survival outcomes specifically for IP-SNSCC patients [36, 37]. Due to the limitations of these studies, the prognostic value of HPV in SNSCC requires additional studies with careful attention to tumor type and larger sample sizes. Recently, using a large cohort from the National Cancer Database, Suat et al. found that HPV was a favorable prognostic factor in SNSCC [18]. However, the selection bias and dissimilar HPV testing in the database may confound this conclusion. Further studies are required to determine if HPV is a favorable prognostic factor in SNSCC.

## Conclusion

In conclusion, immunostaining for p16 is not a reliable surrogate marker of HPV status in SNSCC. The presence of HPV is rarely detected in DN-SNSCC patients in Eastern China. We found that the presence of HPV DNA was significantly higher in IP-SNSCC patients than in DN-SNSCC patients. However, IP-SNSCC patients frequently lack of p16 overexpression despite the presence of high-risk HPV DNA.

## Data Availability

The datasets used during the current study are available from the corresponding author on reasonable request.

## Abbreviations

DN-SNSCC: de-novo sinonasal squamous cell carcinoma
IP-SNSCC: inverted papilloma derived sinonasal squamous cell carcinoma
HPV: human papillomavirus
SCC: squamous cell carcinomas
FFPE: Formalin-fixed and paraffin-embedded

## References

[1] Marur S, D'Souza G, Westra WH, Forastiere AA (2010). HPV-associated head and neck cancer: a virus-related cancer epidemic. Lancet Oncol.

[2] Mourad M, Jetmore T, Jategaonkar AA, Moubayed S, Moshier E, Urken ML (2017). Epidemiological trends of head and neck Cancer in the United States: a SEER population study. J Oral Maxillofac Surg.

[3] Husain N, Neyaz A (2017). Human papillomavirus associated head and neck squamous cell carcinoma: controversies and new concepts. J Oral Biol Craniofac Res.

[4] Isayeva T, Li Y, Maswahu D, Brandwein-Gensler M (2012). Human papillomavirus in non-oropharyngeal head and neck cancers: a systematic literature review. Head Neck Pathol.

[5] Syrjanen K, Syrjanen S (2013). Detection of human papillomavirus in sinonasal carcinoma: systematic review and meta-analysis. Hum Pathol.

[6] Gillison ML, Castellsague X, Chaturvedi A (2014). Eurogin roadmap: comparative epidemiology of HPV infection and associated cancers of the head and neck and cervix. Int J Cancer.

[7] Karligkiotis A, Lepera D, Volpi L (2016). Survival outcomes after endoscopic resection for sinonasal squamous cell carcinoma arising on inverted papilloma. Head Neck.

[8] von Buchwald C, Bradley PJ (2007). Risks of malignancy in inverted papilloma of the nose and paranasal sinuses. Curr Opin Otolaryngol Head Neck Surg.

[9] Zhao RW, Guo ZQ, Zhang RX (2016). Human papillomavirus infection and the malignant transformation of sinonasal inverted papilloma: a meta-analysis. J Clin Virol.

[10] Arndt O, Nottelmann K, Brock J, Neumann OG (1994). Inverted papilloma and its association with human papillomavirus (HPV). A study with polymerase chain reaction (PCR). HNO.

[11] Fakhry C, Lacchetti C, Perez-Ordonez B (2018). Human papillomavirus testing in head and neck carcinomas: ASCO clinical practice guideline endorsement summary of the CAP guideline. J Oncol Pract.

[12] Liu SS, Leung RC, Chan KK, Cheung AN, Ngan HY (2010). Evaluation of a newly developed GenoArray human papillomavirus (HPV) genotyping assay and comparison with the Roche linear Array HPV genotyping assay. J Clin Microbiol.

[13] Mirghani H, Casiraghi O, Amen F (2015). Diagnosis of HPV-driven head and neck cancer with a single test in routine clinical practice. Mod Pathol.

[14] Sano D, Oridate N (2016). The molecular mechanism of human papillomavirus-induced carcinogenesis in head and neck squamous cell carcinoma. Int J Clin Oncol.

[15] Smeets SJ, Hesselink AT, Speel EJ (2007). A novel algorithm for reliable detection of human papillomavirus in paraffin embedded head and neck cancer specimen. Int J Cancer.

[16] Bishop JA, Guo TW, Smith DF (2013). Human papillomavirus-related carcinomas of the sinonasal tract. Am J Surg Pathol.

[17] Alos L, Moyano S, Nadal A (2009). Human papillomaviruses are identified in a subgroup of sinonasal squamous cell carcinomas with favorable outcome. Cancer-Am Cancer SOC.

[18] Kilic S, Kilic SS, Kim ES (2017). Significance of human papillomavirus positivity in sinonasal squamous cell carcinoma. Int Forum Allergy Rhinol.

[19] Pezzuto F, Buonaguro L, Caponigro F (2015). Update on head and neck Cancer: current knowledge on epidemiology, risk factors, molecular features and novel therapies. Oncology.

[20] Young D, Xiao CC, Murphy B, Moore M, Fakhry C, Day TA (2015). Increase in head and neck cancer in younger patients due to human papillomavirus (HPV). Oral Oncol.

[21] Doxtader EE, Katzenstein AL (2012). The relationship between p16 expression and high-risk human papillomavirus infection in squamous cell carcinomas from sites other than uterine cervix: a study of 137 cases. Hum Pathol.

[22] Vitzthum LK, Mell LK (2018). The role of p16 as a biomarker in nonoropharyngeal head and neck cancer. Oncotarget.

[23] Bussu F, Sali M, Gallus R (2013). HPV infection in squamous cell carcinomas arising from different mucosal sites of the head and neck region. Is p16 immunohistochemistry a reliable surrogate marker?. Br J Cancer.

[24] Belobrov S, Cornall AM, Young RJ (2018). The role of human papillomavirus in p16-positive oral cancers. J Oral Pathol Med.

[25] Lechner M, Chakravarthy AR, Walter V (2018). Frequent HPV-independent p16/INK4A overexpression in head and neck cancer. Oral Oncol.

[26] Sahnane N, Ottini G, Turri-Zanoni M (2019). Comprehensive analysis of HPV infection, EGFR exon 20 mutations and LINE1 hypomethylation as risk factors for malignant transformation of sinonasal-inverted papilloma to squamous cell carcinoma. Int J Cancer.

[27] LaPak KM, Burd CE (2014). The molecular balancing act of p16(INK4a) in cancer and aging. Mol Cancer Res.

[28] Chung CH, Zhang Q, Kong CS (2014). p16 protein expression and human papillomavirus status as prognostic biomarkers of nonoropharyngeal head and neck squamous cell carcinoma. J Clin Oncol.

[29] Zhao N, Ang MK, Yin XY (2012). Different cellular p16(INK4a) localisation may signal different survival outcomes in head and neck cancer. Br J Cancer.

[30] Craig SG, Anderson LA, Schache AG (2019). Recommendations for determining HPV status in patients with oropharyngeal cancers under TNM8 guidelines: a two-tier approach. Br J Cancer.

[31] Mohajeri S, Lai C, Purgina B (2018). Human papillomavirus: an unlikely etiologic factor in sinonasal inverted papilloma. LARYNGOSCOPE.

[32] Wang Huan, Zhai Changwen, Liu Juan, Wang Jingjing, Sun Xicai, Hu Li, Wang Dehui (2019). Low prevalence of human papillomavirus infection in sinonasal inverted papilloma and oncocytic papilloma. Virchows Archiv.

[33] Lewis JJ, Westra WH, Thompson LD (2014). The sinonasal tract: another potential "hot spot" for carcinomas with transcriptionally-active human papillomavirus. Head Neck Pathol.

[34] Chowdhury N, Alvi S, Kimura K (2017). Outcomes of HPV-related nasal squamous cell carcinoma. Laryngoscope.

[35] Laco J, Sieglova K, Vosmikova H (2015). The presence of high-risk human papillomavirus (HPV) E6/E7 mRNA transcripts in a subset of sinonasal carcinomas is evidence of involvement of HPV in its etiopathogenesis. Virchows Arch.

[36] Yan CH, Newman JG, Kennedy DW, Palmer JN, Adappa ND (2017). Clinical outcomes of sinonasal squamous cell carcinomas based on tumor etiology. Int Forum Allergy Rhinol.

[37] Choi JW, Kim SG, Kim YM, Yoon YH, Kim AY, Rha KS (2012). Clinical and histologic features of inverted papilloma-associated malignancy. Eur Arch Otorhinolaryngol.

